# Thysanoptera (Thrips) Within Citrus Orchards in Florida: Species Distribution, Relative and Seasonal Abundance Within Trees, and Species on Vines and Ground Cover Plants

**DOI:** 10.1673/031.006.4501

**Published:** 2006-12-05

**Authors:** Carl C. Childers, Sueo Nakahara

**Affiliations:** ^1^ University of Florida, Department of Entomology and Nematology, Citrus Research and Education Center, 700 Experiment Station Road, Lake Alfred, FL 33850; ^2^ USDA, ARS, Systematic Entomology Laboratory, 10300 Baltimore Blvd, Bldg 005, Room 137, Beltsville, MD 20705

**Keywords:** Thrips, Terebrantia, Phlaeothripinae, Idolothripinae, Thripidae, Phlaeothripidae

## Abstract

Seven citrus orchards on reduced to no pesticide spray programs were sampled for Thysanoptera in central and south central Florida. Inner and outer canopy leaves, fruits, twigs, trunk scrapings, vines and ground cover plants were sampled monthly between January 1995 and January 1996. Thirty-six species of thrips were identified from 2,979 specimens collected from within citrus tree canopies and 18,266 specimens from vines and ground cover plants within the seven citrus orchards. The thrips species included seven predators [*Aleurodothrips fasciapennis* (Franklin), *Karnyothrips flavipes* (Jones), *K*. *melaleucus* (Bagnall), *Leptothrips cassiae* (Watson), *L*. *macroocellatus* (Watson), *L*. *pini* (Watson), and *Scolothrips sexmaculatus* (Pergande)] 21 plant feeding species [*Anaphothrips n*. sp., *Arorathrips mexicanus* (Crawford), *Aurantothrips orchidaceous* (Bagnall), *Baileyothrips limbatus* (Hood), *Chaetanaphothrips orchidii* (Moulton), *Danothrips trifasciatus* (Sakimura), *Echinothrips americanus* (Morgan), *Frankliniella bispinosa* (Morgan), *F*. *cephalica* (Crawford), *F*. *fusca* (Hinds), *F*. *gossypiana* (Hood), *Frankliniella* sp. (runneri group), *Haplothrips gowdeyi* (Franklin), *Heliothrips haemorrhoidalis* (Bouché), *Leucothrips piercei* (Morgan), *Microcephalothrips abdominalis* (Crawford), *Neohydatothrips floridanus* (Watson), *N*. *portoricensis* (Morgan), *Pseudothrips inequalis* (Beach), *Scirtothrips* sp., and *Thrips hawaiiensis* (Morgan)]; and eight fungivorous feeding species [*Adraneothrips decorus* (Hood), *Hoplandrothrips pergandei* (Hinds), *Idolothripinae* sp., *Merothrips floridensis* (Watson), *M*. *morgani* (Hood), *Neurothrips magnafemoralis* (Hinds), *Stephanothrips occidentalis* Hood and Williams, and *Symphyothrips* sp.]. Only *F*. *bispinosa*, *C*. *orchidii*, *D*. *trifasciatus*, and *H*. *haemorrhoidalis* have been considered economic pests on Florida citrus. *Scirtothrips* sp. and *T*. *hawaiiensis* were recovered in low numbers within Florida citrus orchards. Both are potential pest species to citrus and possibly other crops in Florida. The five most abundant thrips species collected within citrus tree canopies were: *A*. *fasciapennis*, *F*. *bispinosa*, *C*. *orchidii*, *K. flavipes*, and *D*. *trifasciatus*. In comparison, the following five thrips species were most abundant on vines or ground cover plants: *F*. *bispinosa*, *H*. *gowdeyi*, *F*. *cephalica*, *M*. *abdominalis*, and *F*. *gossypiana*. Fifty-eight species of vines or ground cover plants in 26 families were infested with one or more of 27 species of thrips.

## Introduction

The Thysanoptera (thrips) exist in a wide array of habitats. Many species are serious economic pests of various crops (Lewis 1997). Several thrips species are important pests of citrus including citrus thrips, *Scirtothrips citri* (Moulton) in California and Arizona and *S*. *aurantii* Faure in South Africa (Bedford 1943; Talhouk 1975; Tanigoshi and Nishio-Wong 1982). Feeding injury by these species results in scarring of rind tissue in a fairly uniform ring encircling the stem end of the fruit. Navel oranges are a preferred host of citrus thrips in California and subsequent rind blemish injury results in the rejection of fruit for the fresh market. Young twigs, leaves, and leaf buds are also fed upon by citrus thrips resulting in non-economic types of injury to the trees (Jeppson 1989).

*Frankliniella bispinosa* (Morgan) and *F*. *kelliae* Sakimura in Florida cause premature flower drop in navel and ‘Valencia’ oranges as a result of adult and larval feeding during bloom (Childers and Achor 1991; Childers 1992). *Chaetanaphothrips orchidii* (Moulton), *Danothrips trifasciatus* Sakimura, and *Heliothrips haemorrhoidalis* (Bouché) were found to cause rind blemish damage (ring spot) on red grapefruit varieties in Florida (Childers and Frantz 1994).

**Table 1.**
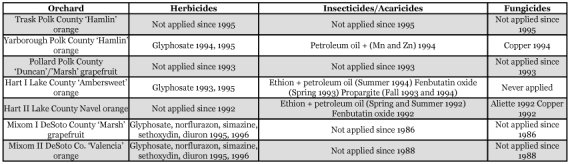
Pesticide spray programs for seven citrus orchards sampled for Thysanopteran species in Florida during 1995–1996.

Eighteen species were identified on citrus flowers and *F*. *bispinosa* was the prevalent species (Childers et al. 1990, Childers and Beshear 1992). In a later study, 86 species of thrips were collected with sticky card traps that were placed within Florida citrus orchards at several locations (Childers et al. 1998). However, only limited information was available as to which of these species were actual inhabitants of Florida citrus orchards. Development of effective management strategies for thrips pests on Florida citrus requires an understanding of their respective biologies including their relative abundance, associated predators, and distributions within the orchards. Therefore, this study was initiated over a 13-month interval to determine the species complex of Thysanoptera that occur within selected Florida citrus orchards as well as the thrips present on associated vine and ground cover plants within those orchard sites.

## Materials and Methods

**Figure 1.**
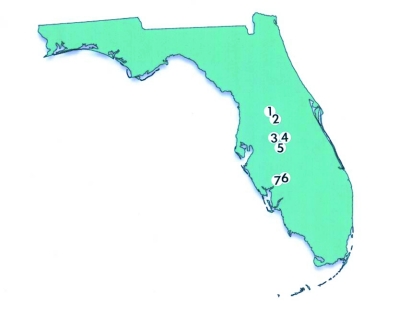
Map of Florida showing the locations of the seven citrus orchard sites: 1 - Hart I, 2 - Hart II, 3 - Pollard, 4 - Trask, 5 - Yarborough, 6 - Mixom I, 7 - Mixom II.

Seven citrus orchards in Polk, Lake, and DeSoto Counties in Central and South-Central Florida on reduced to no pesticide spray programs were sampled monthly over a 13-month interval between January 1995 and January 1996 for Thysanoptera (Table 1). The Trask, Pollard, and Yarborough orchards were located in the Highlands City vicinity in Polk County all within 10 km of each other. The two Hart orchards were about 5 km apart and located immediately off County Road 469 about 10 km northwest of Mascotte in Lake County. The two Mixom orchards were located in southeastern Arcadia in DeSoto County about 10 km apart (Figure 1).

Thrips species were collected separately from (1) 100 inner leaves (2) 100 outer leaves, (3) 10–20 twigs, (4) 25-immature fruits, and (5) individual mature fruit samples. Tree trunk scrapings (6) were collected into a 5-liter bucket containing about 250 ml of 80% ethanol. A stiff brush was used to make 15–20 firm, short downward strokes on the surface of the main trunk and scaffold limbs of each citrus tree sampled and scrapings were directed into a bucket placed directly below to collect the arthropods. Loose bark or debris and lichens were also collected from the tree trunks. Each of the six sample types was collected individually and replicated six to eight times. Many thrips species would rapidly leave a disturbed leaf or fruit. Therefore, rapid preservation of the thrips fauna was intended to accurately measure what was present within the sample.

Individual fruit, leaves, and twigs were collected and dropped immediately into a bucket containing about 250 ml of 80% ethanol, and vigorously agitated in the solution. Most of the fruit, leaf, or twig samples were discarded on site and the alcohol wash was transferred into a labeled glass jar and returned to the laboratory for processing.

Ground cover plants or vines were also sampled from five of the seven orchard sites. Selection of plants varied at each location and depended upon their prevalence. The two Mixom sites had been treated with herbicide and lacked ground cover plants for sampling (Table 1). Plant material for each species included leaves, stems, and flowers (when present), and a single sample was taken from one or more plants for each selected plant species present per orchard site on a given date. A sufficient volume of a ground cover plant or vine species was gathered to fill a 473 ml glass jar containing 50–100 ml of 80% ethanol to near capacity. Each jar containing one plant species was returned to the laboratory for processing. A representative sample of each plant was also collected and placed in a plant press in the field between sheets of newspaper for eventual identification. Plant identifications to species were completed by Kent Perkins at the Herbarium, University of Florida, Gainesville. Collection data were included with samples in both jars and the plant press which included location, date, and code (e.g., weed A, B, C).

Each thrips species in each processed sample was recorded and then placed into a labeled vial containing 80% ethanol. Thrips were later removed, and individually slide-mounted in Hoyer's mounting medium (Krantz 1978) then oven-cured for at least three weeks at 45–47°C. Numerous thrips (more than 500) were present in many of the ground cover plant samples so subsamples from these were taken by proportionally selecting between 30 and 100 representative specimens by color, sex, or larval size.

## Results and Discussion

### Thrips Species Composition

Thirty-six species of thrips were identified from 2,979 specimens collected within citrus tree canopies and 18,266 specimens from vines or ground cover plants (Tables 2, 3). Ten thrips species were recovered only from within citrus tree canopies and included: *Merothrips floridensis* (Watson), M. *morgani* (Hood), *Frankliniella* sp. (runneri group), *Heliothrips haemorrhoidalis* (Bouché), *Adraneothrips decorus* (Hood), *Hoplandrothrips pergandei* (Hinds), Idolothripinae, *Neurothrips magnafemoralis* (Hinds), *Stephanothrips occidentalis* (Hood and Williams), and *Symphyothrips* sp. Fourteen thrips species were recovered from both citrus tree canopies and vine or ground cover plants and included: *Anaphothrips* n. sp., *Arorathrips mexicanus* (Crawford), *Chaetanaphothrips orchidii* (Moulton), *Danothrips trifasciatus* (Sakimura), *Frankliniella bispinosa* (Morgan), *F*. *cephalica* (Crawford), *Microcephalothrips abdominalis* (Crawford), *Scirtothrips* sp., *Aleurodothrips fasciapennis* (Franklin), *Haplothrips gowdeyi* (Franklin), *Karnyothrips flavipes* (Jones), *K*. *melaleucus* (Bagnall), *Leptothrips macroocellatus* (Watson), and *Leucothrips piercei* (Morgan). The remaining twelve species were collected only from either vine or ground cover plants and included: *Aurantothrips orchidaceous* (Bagnall), *Baileyothrips limbatus* (Hood), *Echinothrips americanus* (Morgan), *Frankliniella fusca* (Hinds), *F*. *gossypiana* (Hood), *Neohydatothrips floridanus* (Watson), *N*. *portoricensis* (Morgan), *Pseudothrips inequalis* (Beach), *Scolothrips sexmaculatus* (Pergande), *Thrips hawaiiensis* (Morgan), *Leptothrips cassiae* (Watson), and *L*. *pini* (Watson) (Tables 2,3). Among the 36 thrips species were seven predacious, 21 phytophagous and eight fungal feeding species. Four of the phytophagous species are known pests of Florida citrus: *F*. *bispinosa*, *C*. *orchidii*, *D*. *trifasciatus*, and *H*. *haemorrhoidalis* (Childers 1992, 1999; Childers and Frantz 1994). Two additional phytophagous species, *Scirtothnps* sp. and *T*. *hawaiiensis* are potential economic pests of Florida citrus that require close monitoring over the coming years. The remaining 23 phytophagous and fungal feeding thrips species are not economic pests of citrus.

### Distribution, relative and seasonal abundance of each thrips species on citrus and on vine and ground cover plants within citrus orchards

The five most abundant thrips species found within citrus tree canopies were: *A*. *fasciapennis* (1,015), *F*. *bispinosa* (680), *C*. *orchidii* (360), *K*. *flavipes* (206), and *D*. *trifasciatus* (156). In comparison, the five most abundant species found on vines or ground cover plants were: *F*. *bispinosa* (6,151), *H*. *gowdeyi* (5,003), *F*. *cephalica* (Crawford) (4,310), *M*. *abdominalis* (1,036), and *F*. *gossypiana* (Hood) (207).

Each of the 36 species is divided into three groups below. Their distributions within citrus trees or from one or more of 58 species of plants, vines, and groundcover representing 26 families are presented. The thrips species associations and dates collected from each of these plants are shown in Table 3.

### Predacious species

#### *Aleurodothrips fasciapennis* (Franklin)

This species was the most abundant predacious thrips collected within the citrus tree canopy during this study (Tables 2, 3). *A*. *fasciapennis* was present in all seven orchard sites on leaves, fruit and twigs. This species was more abundant on inner versus outer leaf samples with 495 and 171 individuals, respectively (Table 4).

*A*. *fasciapennis* was found during every month sampled and most abundant on inner leaves during the months of March and April 1995 (Table 4). This was similar to results from a previous study by Selhime et al. (1963) who found this species to be most abundant during spring and early summer. *A*. *fasciapennis* was rarely found on ground cover plants with a total of three adults collected from *Richardia brasiliensis*, and *Lantana* camera in August and on *Amaranthus spinosus* in the Pollard site in October (Table 3).

**Table 2.**
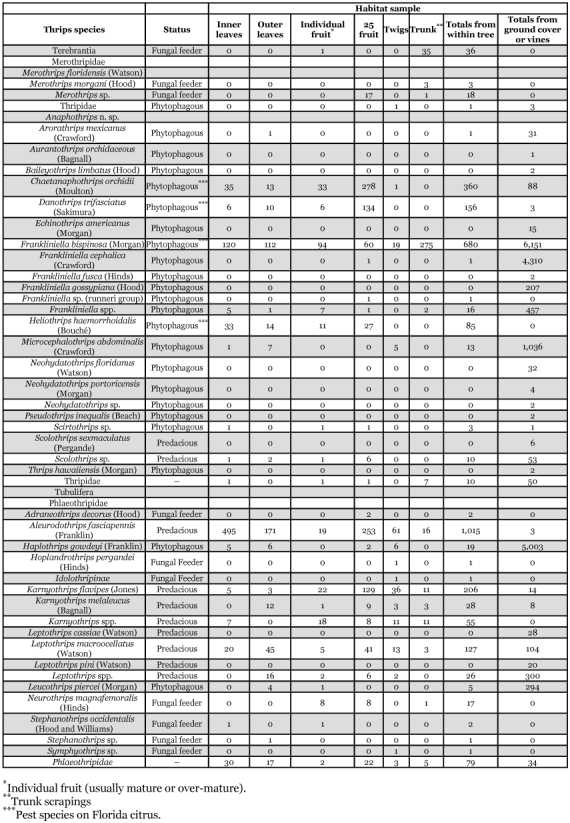
Distribution and comparative numbers of Thysanopteran species collected within seven Florida citrus orchards between January 1995 and January 1996.

**Table 3.**
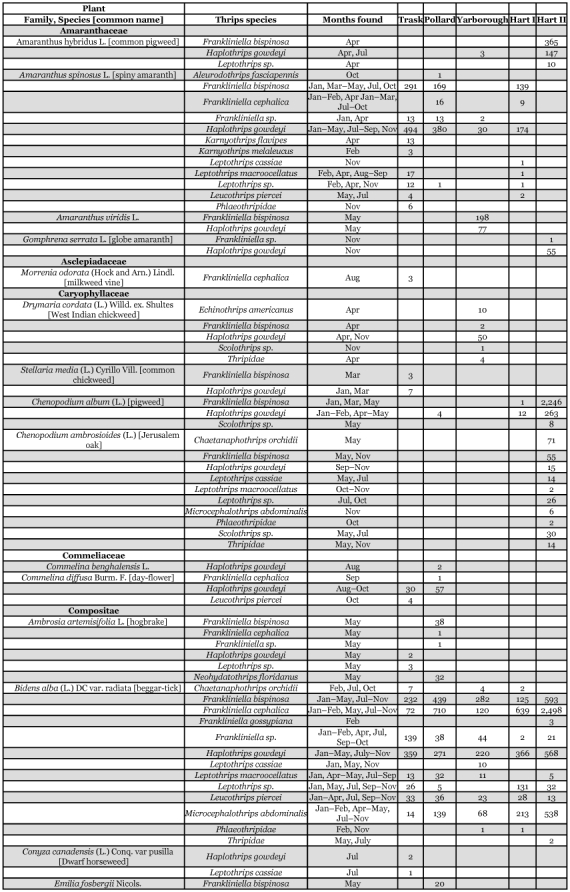
Thrips species collected from selected vine and ground cover plants within seven Florida citrus orchards between January 1995 and January 1996.

**Continued:**
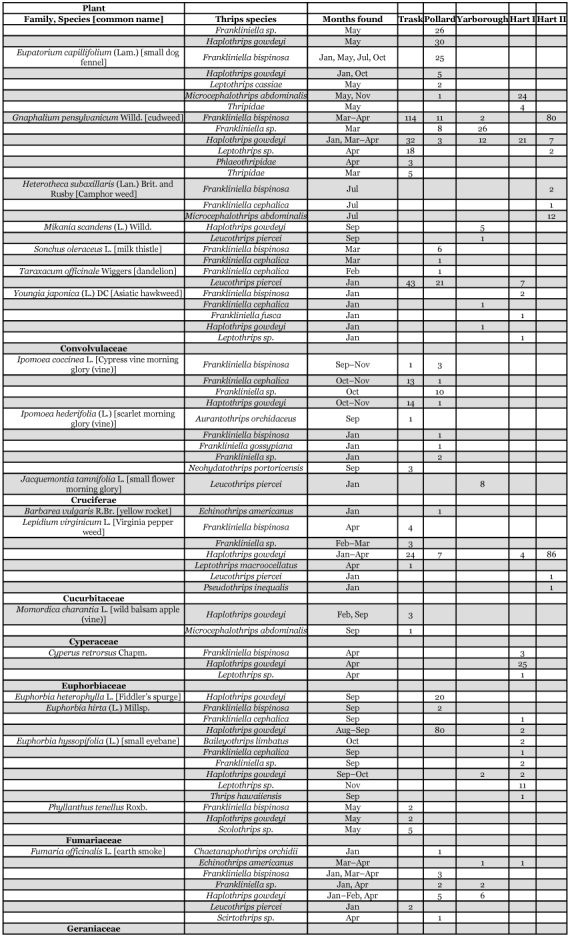

**Continued:**
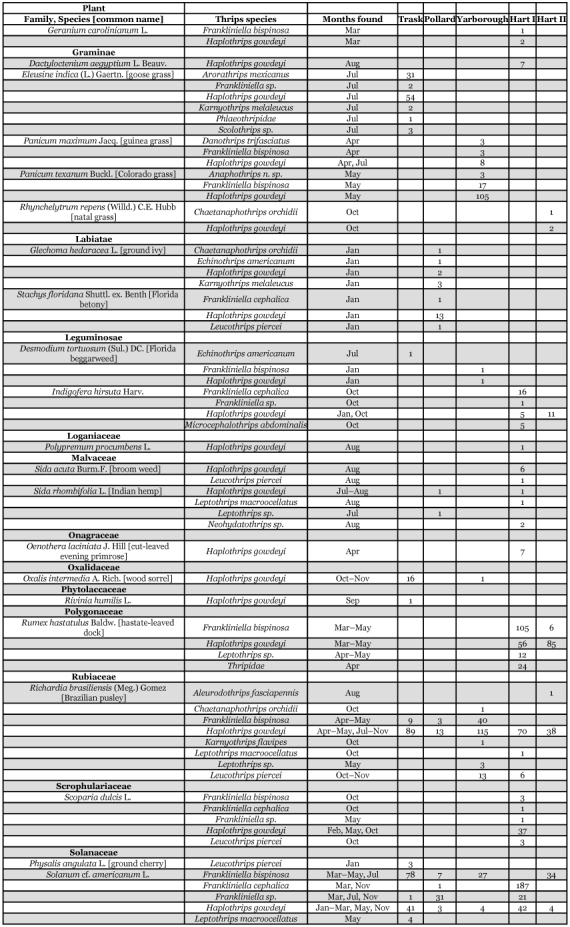

**Continued:**
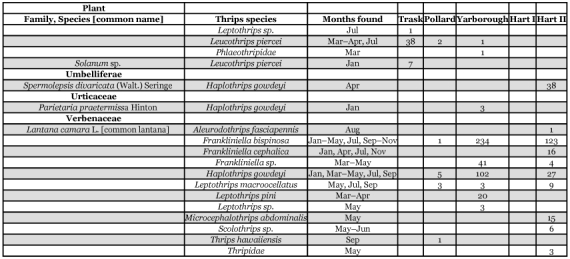

*A*. *fasciapennis* will feed and develop on purple scale, *Lepidosaphes beckii* (Newman), Florida red scale, *Chrysomphalus aonidum* (L.), chaff scale, *Parlatoria pergandii* Comstock, oleander scale, *Aspidiotus nerii* Bouché (Beshear 1975), and mango scale, *Aulacaspis tubercularis* (Newstead) (Labuschagne et al. 1995); cloudy-winged whitefly, *Dialeurodes citrifolii* (Morgan); six-spotted mite, *Eotetranychus sexmaculatus* (Riley), and citrus red mite, *Panonychus citri* (McGregor) (Selhime et al. 1963; Watson et al. 1998). Beattie (1985) found that *A*. *fasciapennis* played a major role in the suppression of California red scale, *Aonidiella aurantii* (Maskell) on citrus in China.

#### *Karnyothrips flavipes* (Jones)

This was the second most abundant predacious thrips species with 94% collected from within citrus tree canopies (Tables 2,3). *K*. *flavipes* is a predator of several species of pit scales *Asterolecanium* spp., armored scale in *Parlatoria* spp., and *Pseudaonidia duplex* (Cockerell), soft scales (*Saissetia* spp.), whiteflies and mites that infest citrus trees and other plants (Pitkin 1976).

#### *Kamyothrips melaleucus* (Bagnall)

This species is a predator of soft scales (Pitkin 1976). Collection data are presented in Tables 2 and 3.

#### *Karnyothrips* spp

55 larvae were collected from leaves, fruits, twigs, and trunk scrapings within citrus tree canopies (Table 2). No larvae were collected from vine or ground cover plants.

#### *Leptothrips cassiae* (Watson)

This species was collected only from the following ground cover plants: *A*. *spinosus, Chenopodium ambrosioides*, *Bidens alba, Conyza canadensis*, and *Eupatorium capillifolium* (Tables 2, 3).

#### *Leptothrips macroocellatus* (Watson)

This was the most common species of *Leptothrips* collected from January through May, and July through November (Table 2). Generally, populations were low (1–2) although 11 adults were collected from a fruit sample on May 15. Adult *L*. *macroocellatus* were collected from *A*. *spinosus*, *C*. *ambrosioides*, *B*. *alba*, *Lepidium virginicum*, *Sida rhombifolia*, *R*. *brasiliensis*, *Solanum americanum*, and *L*. *camara* (Table 3).

#### *Leptothrips pini* (Watson)

Twenty females were collected from a single sample of *L*. *camara* in Yarborough on March 30, 1995 (Tables 2, 3). None were collected from within citrus tree canopies.

#### *Leptothrips* spp

Adults and larvae of all *Leptothrips* species combined were the second most abundant group of predacious thrips after *A*. *fasciapennis* (Table 2). Sixty-five percent of the *Leptothrips* larvae collected on ground cover plants were on *B*. *alba* (Table 3).

#### *Scolothrips sexmaculatus* (Pergande)

Both adults and larvae of *S*. *sexmaculatus* feed on *P*. *citri*, *Tetranychus urticae* Koch, *Eotetranychus* *banksi* (Riley), *Bryobia praetiosa* Koch, and several other spider mite species as well as the citrus rust mite, *Phyllocoptruta oleivora* (Ashmead) (Bailey 1939).

**Table 4.**
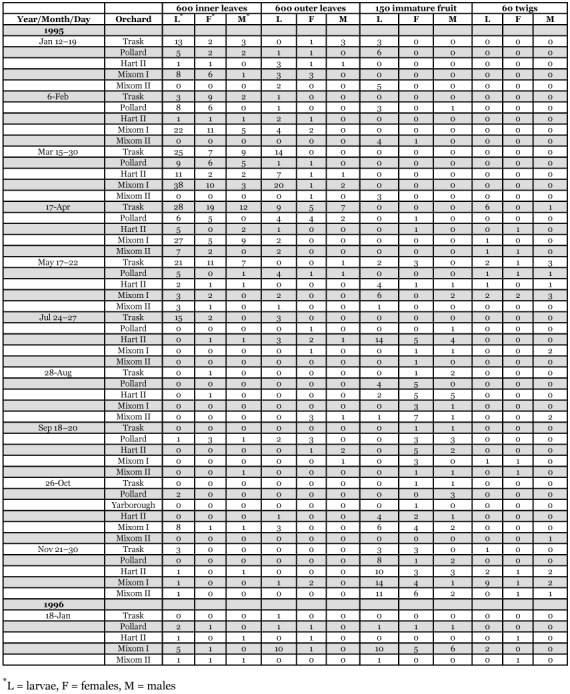
Abundance and distribution of *Aleurodothrips fasciapennis* on Florida citrus trees.

#### *Scolothrips* spp

Collection data for *Scolothrips* spp. are given in Table 3. *Scolothrips* spp. occurred on fruit in April and July with a maximum of 6 larvae collected at Hart II in July and from outer leaf samples in May at two locations. *Scolothrips pallidus* (Beach) has been previously collected from Florida citrus orchards (Childers et al., 1994).

### Plant feeders

#### *Anaphothrips* n. sp

Collection data are presented in Tables 2 and 3. Nakahara (1995) reported 17 Nearctic species in the genus *Anaphothrips* associated with Gramineae.

#### *Arorathrips mexicanus* (Crawford)

A single female was collected on outer leaves at Hart I on October 2, 1995 (Table 2). 25 males, 3 females, and 3 associated larvae were collected from *Eleusine indica* on July 24, 1995 at Trask (Tables 2,3).

#### *Aurantothrips orchidaceous* (Bagnall)

A single female was collected from the vine, *Ipoemoea hederifolia* on September 18, 1995 at Trask (Tables 2, 3). This thrips is found on orchids (Sakimura, 1967).

#### *Baileyothrips limbatus* (Hood)

Collection data are presented in Tables 2 and 3. This species was collected on *Euphorbia* spp. for the first time in Florida and the continental United States by Frantz (1993) in Palm Beach County during August. Sakimura (1986) collected *B*. *limbatus* on *Desmodium* sp. in Jamaica.

#### *Chaetanaphothrips orchidii* (Moulton)

448 females and immatures were recorded from the seven orchard sites with 360 collected within citrus tree canopies and 88 from vine or ground cover plants (Tables 2, 3). This species was most abundant in two grapefruit orchards (Pollard and Mixom I sites) on fruits and only a few were taken from inner and outer leaves or twigs (Table 4). *Chaetanaphothrips orchidii* was collected from ground cover plants: *B*. *alba* in February, October, and November; *Glechoma hedaracea* in October; *Fumaria officinalis* in January; *R. braziliensis* in October; *C*. *ambrosioides* in May and *Rhynchelytrum repens* in October. 71 of the 88 *C*. *orchidii* were collected from *C*. *ambrosioides* (Table 4). Other larval host plants included: *Begonia*, *Emilia*, *Ephiphyllum*, *Hemierliodendron*, *Hypoxis*, *Musa*, and *Tradescantia* (Morison 1957). Known hosts of *C*. *orchidii* include: *Alternanthera*, *Amaranthus*, *Anthurium androeanum*, *Bougainvillea*, *Chrysanthemum*, *Cereus*, *Cyclamen*, *Cyrtandra* sp., *Tradescantia zebrina, Zea mays*, *Petroselinum crispum*, *Cryptotaenia japonica, Euphorbia* sp., *Hedytois* sp., *Ipomoea alba*, *I*. *congesta*, *Lycopersicon* sp., *Monstera*, *Philodendron*, *Pisonia*, *Rhododendron simsii*, *Sonchus oleraceus*, *Spathoglottis plicata*, and *Zingiber zerumbet* (Kara et al. 1987; Mantel and van de Vrie 1988). In addition, adults and larvae have been collected on various grass species including: *Coix lacryma-jobi*, *Digitana pruriens*, *Panicum purpurascens*, *Paspalum conjugatum*, *P*. *orbiculare*, and *Trichachne insularis* in wet areas.

**Figure 2.**
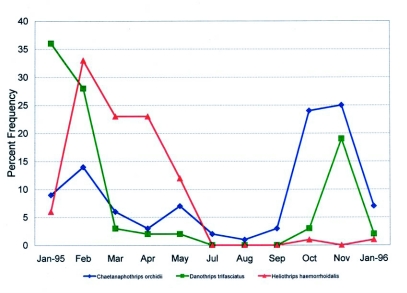
Seasonal frequency distributions of three phytophagous pest thrips species on Florida citrus in seven citrus orchard sites in central and south-central Florida during 1995–1996.

Thompson (1939) first reported *C*. *orchidii* as a pest on grapefruit in Florida. The orchid thrips is one of three species in Florida that feed where clustered fruit begin to touch, beginning in early May. Mostly red grapefruit varieties, and to a lesser extent white grapefruit, and occasionally round orange varieties such as ‘Valencia’ or ‘Hamlin’, are affected by *C*. *orchidii*, *D*. *trifasciatus*, and the greenhouse thrips, *H*. *haemorrhoidalis*. Damage resulting from their feeding can occur from onset of grapefruit beginning to touch until the fruit are harvested (Childers and Frantz 1994). This survey found 448 *C*. *orchidii* (65%), 159 *D*. *trifasciatus* (23%), and 85 *H*. *haemorrhoidalis* (12 %). *C*. *orchidii* was present throughout the season in the citrus orchards and most abundant during the fall months of October and November (Figure 2). Re-infestation of maturing clustered citrus fruits can occur with movement of this thrips pest from alternate hosts including many weed species occurring within citrus orchards to maturing clustered fruits throughout the season.

This thrips severely damaged *Anthurium* sp. in a greenhouse in Apopka, Orange County, Florida during December (Osborne 1993) and infested *Maranta leuconeura* var. *erythroneura* in the same area during April (Wilber and Capitano 1998).

#### *Danothrips trifasciatus* (Sakimura)

Collection data are presented in Tables 2 and 3. Most specimens were collected in the Pollard orchard on fruit samples with only a few individuals collected on leaves in the Pollard, Hart I, Mixom I and II sites. This species was most abundant during January and February in the citrus orchards and toward the end of harvest for grapefruit varieties in Florida (Fig. 2). This species was collected for the first time within the continental United States during earlier sampling in south Florida on red grapefruit varieties (Childers and Frantz 1994).

This species feeds on flowers and leaves of various plants including: *P*. *crispum*, *Bougainvillea*, *Z*. *zerumbet*, *Alpinia purpurea*, *Anthurium andreanum*, *Paspalum orbiculare*, *P*. *conjugatum*, *I*. *alba*, *Costus*, *Melicoccus bijugatus*, young Z. mays leaves, and banana (Sakimura 1975; Bhatti 1980).

#### *Echinothrips americanus* (Morgan)

*E*. *americanus* (Table 3) is a polyphagous, leaf-feeding thrips found on at least 40 cultivated and 59 native host plant species (Getting et al. 1993). Frantz and Mellinger (1990) found this thrips species on *Chrysanthemum* during July in Florida between 1986 and 1990. Additional Florida records included coralberry, *Ardisia crenata* in Sorrento, Lake County in November (Murphy 1994) and *Mimosa pudica* in Brooksville, Hernando County in July (Dudley 1994).

#### *Frankliniella bispinosa* (Morgan)

680 specimens were collected from inner and outer leaves, fruit, and twigs within citrus canopies in low numbers throughout the year compared with 6,151 collected from 31 vine and ground cover plant species (Tables 2, 3).

Numerous *F*. *bispinosa* larvae and associated pupae were present on the main trunks and scaffold branches of citrus trees in Florida during February and March (Tables 2, 5). Also, large numbers of second instar larvae drop to the ground and pupate (Childers et al. 1994). Comparable results were shown by Grout et al. (1986) with *S*. *citri* in citrus orchards in California where a percentage of the population pupated within or near the surface of the soil in leaf litter and a percentage pupated within the citrus trees.

*F*. *bispinosa* was the most abundant thrips species recorded on 31 vines and ground cover plant species within 5 of the 7 orchard sites (Table 3). The remaining 2 groves were on herbicide programs and void of ground plant cover (Table 1). Two weed species alone [*Chenopodium album* (L.) and *B*. *alba* (L.)] accounted for 36% and 27%, respectively, of *F*. *bispinosa* collected from vines or ground cover plants. This suggests that the composition of ground cover plants within different citrus orchards could contribute to grove population differences of this pest thrips species. *F*. *bispinosa* was present every month of the year on one or more vine or ground cover plants sampled between January 1995 and January 1996 (Table 6) with general trends of higher larval and adult thrips populations occurring during April and again in November. Multiple, overlapping generations occur within vines and ground cover plants as well as within citrus tree canopies throughout the year. These hosts combined with a larger, more diverse host plant range demonstrates why *F*. *bispinosa* occurs in such abundance throughout Florida and how it has the potential to become such a localized pest on several different crops of agricultural or horticultural importance.

**Table 5.**
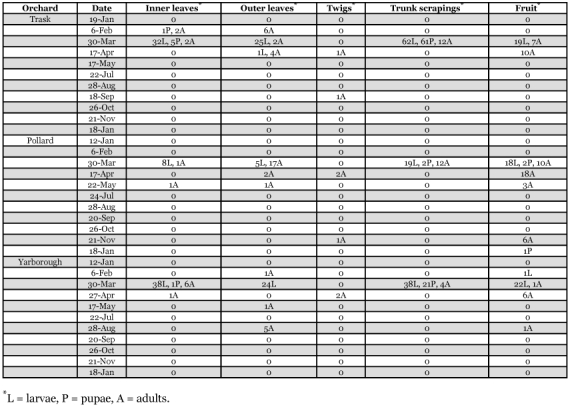
Seasonal distribution of *Frankliniella bispinosa* within trees in three central Florida citrus orchards during 1995–1996.

Adults and larvae of *F*. *bispinosa* have been recorded in numbers as high as 200 per open citrus flower of ‘Rhode Red’ Valencia and ‘Murcott’ oranges during the major bloom period between February and April each year (Childers, unpublished). Generally, a few to 100 *F*. *bispinosa* can be found in the flowers of various citrus varieties during the major blooming period that occurs between February and April (Childers 1999). This species is an important pest of citrus in Florida that causes pre-mature flower drop and reduced yields of navel and Valencia oranges (Childers and Achor 1991; Childers 1992, 1999). Adults and larvae feed on the ovary, style, nectary, petals, and anthers of both swollen buds and open citrus flowers between February and April. Feeding injury results in cellular evacuation, necrosis, plasmolysis, and cellular collapse of floral or bud tissue extending 1 to 5 cells deep. Stress ethylene production occurs resulting in premature flower or bud abortion and reduced fruit set. Preliminary observations indicate that *F*. *bispinosa* can produce rind blemish injuries on developing Murcott, a tangor variety (M. E. Rogers and C. C. Childers, unpublished).

It is also a pest on other crops in Florida including the flowers of Hibiscus, chrysanthemum, snapdragon, bell pepper, black-eyed peas, blueberry, eggplant, corn, cucumber, peanut, watermelon, and juniper; avocado fruits, tomato fruits; and fruit and flowers of strawberry, avocado, and passion fruit (Watson 1922; Fisher and Davenport 1989; Frantz and Mellinger 1990; Mead 1991a, b, c, 1992; 1993). Frantz and Mellinger (1990) found *F*. *bispinosa* on numerous plants including vegetables, ornamentals, trees, and ground cover weed species between February and December in Florida from 1986 to 1990.

#### *Frankliniella cephalica* (Crawford)

A single female was collected from a fruit sample in Trask in October compared with 4,310 specimens collected from ground cover plants (Tables 2, 3, and 6). This species was the third most abundant species found in association with Florida citrus orchards (Table 3). Frantz and Mellinger (1990) recorded *F*. *cephalica* on *Bidens pilosa*, tomato, and mangrove from March through June between 1986 and 1990 in Florida. It is not a pest on Florida citrus and is clearly more of a ground cover inhabitant associated with *B*. *alba* (Childers et al. 1990; Childers and Beshear 1992; Childers et al. 1994).

#### *Frankliniella fusca* (Hinds)

This species was collected only from ground cover plants in Hart I. A single larva was collected from *Youngia japonica* on May 15,1995 and one female from *Spermolepsis divaricata* on April 19, 1995 in Hart I. Frantz and Mellinger (1990) collected *F*. *fusca* adults on *B*. *pilosa*, Chrysanthemum, *Lippia* sp., cucumber, lettuce, grasses, parsley, peanut, pepper, tomato, and hyacinth in Florida between 1986 and 1990.

#### *Frankliniella gossypiana* (Hood)

207 adults and associated larvae of this species were collected in 5 of the orchard sites but only on ground cover plants during January through April and July through November (Table 3).

**Table 6.**
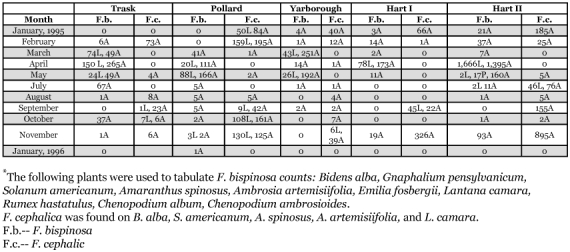
Seasonal and relative abundance of *Frankliniella bispinosa* and *F*. *Cephalic* stages on 12 selected ground cover plants in five citrus orchard sites in central and south-central Florida during January 1995-January 1996.

#### *Frankliniella* sp. (runneri group)

A single male was collected on a ‘Hamlin’ orange fruit on April 17, 1995 in Trask (Table 3).

#### *Frankliniella* spp

16 larvae were collected from citrus leaf and fruit samples compared with 457 larvae from vine or ground cover plants (Tables 2, 3).

#### *Haplothrips gowdeyi* (Franklin)

5022 *H*. *gowdeyi* were collected during this survey. There were 19 adults and larvae collected from within citrus tree canopies compared with 5,022 adults and larvae from 50 vine or ground cover plants (Tables 2, 3). This was the second most abundant thrips found on ground cover plants. *Gnaphilium pensylvanicum* was infested with adults and larvae between January and March. Other infested plants included *Stellaria media* with females only in March, *R*. *brasilensis* with females and larvae in April, May, July, and August through November at the 5 orchard sites not receiving multiple herbicide applications (Tables 1,3). *H*. *gowdeyi* is considered a general flower feeder (Nakahara and Hilburn 1989).

#### *Heliothrips haemorrhoidalis* (Bouché)

85 specimens were collected only from citrus fruits, and inner and outer leaf samples during January through May in Trask, Pollard, Yarborough, and the two Mixom sites (Table 2). The number of thrips per sample was generally low with one or two individuals. No specimens were collected on ground cover plants. This species has a wide host range (Denmark 1985). In Italy, it overwinters in the egg stage but is capable of surviving mild winters (Del Bene et al. 1998). Four generations were produced on Viburnum or myrtle leaves. This is a pest of citrus and capable of causing rind blemish damage on clustered fruit of red grapefruit varieties in Florida (Childers and Stansly, 2005).

#### *Leucothrips piercei* (Morgan)

This species was collected from outer leaves or Hamlin orange fruit in January, March, and April and from 14 ground cover plants throughout the year except the month of August (Tables 2, 3). Frantz and Mellinger (1990) collected *L*. *piercei* from Bok choi in November in Florida.

#### *Microcephalothrips abdominalis* (Crawford)

13 males and females were recovered from inner and outer leaves and twigs between July and November in the Hart I, Hart II, and Mixom II sites compared with 1,036 individuals collected from vine and ground cover plants (Tables 2,3). This species was found in greatest abundance on *B*. *alba*. Frantz and Mellinger (1990) collected specimens on *B*. *pilosa*, Chrysanthemum, *Coreopsis*, Leguminosae, ragweed, and tomato between February and November in Florida from 1986 to 1990. This species was commonly found on Chrysanthemum and *B*. *pilosa* between January and May in Florida. It is a cosmopolitan species that feeds and develops on flowers of Compositae (Bailey 1937).

#### *Neohydatothrips floridanus* (Watson)

This species was collected only from *Ambrosia artemisiifolia* on May 22, 1995 in Pollard (Table 3). It occurs throughout Florida and is not known as a pest.

#### *Neohydatothrips portoricensis* (Morgan)

This species was collected only from *Ipomoea hederifolia* in September at Trask (Table 3). This species has been collected from *I*. *batatas* and *Allium cepa* (Nakahara and Hilburn 1989). Its preferred host is *Ipomoea* spp.

#### *Neohydatothrips* sp

Two larvae were collected from *S*. *rhombifolia* at Hart I during August (Tables 2,3).

#### *Pseudothrips inequalis* (Beach)

Two males were collected only from L. *virginicum* in January at Hart II (Table 3). Other host plants include: *Aster* sp., *Senecio* sp., *Salix nigra*, *Chionanthus virginica*, wild begonia, *Monotrope uniflora*, and *Sparta patens* (Jacot-Guillarmod 1974).

#### *Scirtothrips* sp

One female was collected on a fruit of ‘Marsh’ grapefruit in April at Mixom I. One larva each was recovered from inner leaves and fruit in February at Mixom II and one larva from *F*. *officinalis* in April at Pollard (Tables 2, 3). One male was collected from an emergence cage in a navel orange orchard in Polk County on March 25, 1991 (Childers et al. 1994). Four *Scirtothrips* specimens were collected on Mark V Krome Kote white sticky cards placed in navel orange orchards in southwest Florida between February 9 and May 2, 1990. One female *Scirtothrips* was recovered from a Mark V Krome Kote white sticky card in Polk County on March 22, 1993 and another female from a Chromolux metallic mother-of-pearl Mo5 card on April 9 from the same site (Childers et al. 1998). *Scirtothrips* specimens were never collected from citrus flowers or buds during various experiments between 1988 and 2003 (Childers, unpublished). This species occurred in low numbers in different citrus orchards throughout south and central Florida. An extensive survey of more than 45 vegetable, ornamental, and associated weed plant species was completed between 1986 and 1990 in south Florida by Frantz and Mellinger (1990) and no *Scirtothrips* were collected.

At least four species of *Scirtothrips* (i.e., *S*. *citri* (Moulton), *S*. *aurantii* Faure, *S*. *dorsalis* Hood, and *S*. *dobroskyi* (Moulton) have been collected elsewhere on citrus (Lewis et al. 1997; Hoddle and Mound 2003). The first three species are economic pests of citrus. The species collected in Florida closely resembles *S*. *citri* which was reported in 1986 to occur on grapes in northern Florida (Flowers 1989). However, the species identity remains questionable based on morphological comparisons between series of specimens from Florida and *S*. *citri* from California. Given the importance of some species within this genus, as pests of citrus worldwide, continued close attention to possible future changes in population densities should be given.

#### *Thrips hawaiiensis* (Morgan)

One larva and one female were collected separately from *L*. *camara* in September at Pollard and from *Chamaesyce hyssopifolia* in September at Hart I, respectively (Tables 2,3). *T*. *hawaiiensis* was first reported in Florida, Georgia, and South Carolina after 1967 (Sakimura 1986). This thrips feeds on numerous crops of economic importance worldwide (Sakimura and Nishida 1944; Miyazaki and Kudo 1988; Nakahara and Hilburn 1989). Usually, adults and larvae feed on pollen and cell sap of developing flowers resulting in bud malformation and poor fruit set (Palmer and Wetton 1987). Frantz and Mellinger (1990) recorded this species on cucumber during April between 1986 and 1990 in Florida.

One adult was collected from a Mark V Krome Kote white sticky trap in a navel orange orchard in central Florida during 1991 (Childers et al. 1998). One female was collected from an Olson blue sticky trap (Olson Products, Inc., Medina, Ohio) on March 22, 1993 (Childers et al. 1998). Three females were collected from navel orange flowers in January in Polk County, Florida and one male was collected on catkins of *Salix caroliniana* in Polk County on February 9, 1990 (Childers et al. 1990). This species feeds primarily on the inflorescence of various plants and has been intercepted frequently on cut flowers at United States ports-of-entry (Nakahara 1985).

*T. hawaiiensis* is a serious pest of gladiolus flowers in Taiwan (Chen and Lo 1987). Srivastava and Bhullar (1980) reported *T. hawaiiensis* as a pest on citrus flowers in India. Both larvae and adults reportedly fed on developing flowers with heavily infested blooms failing to set fruit. This species poses a potential future problem for Florida citrus based on such information and continued assessment of its relative abundance and distribution within citrus orchards and ornamental plants is warranted.

### Fungal feeders

#### *Adraneothrips decorus* Hood

Two males were collected from an orange fruit in October at Yarborough (Table 3). 16 specimens were collected from emergence cages in citrus orchards between March 12 and 22 in an earlier study in Florida (Childers et al. 1994). Sakimura (1986) found *A*. *decorus* to be abundant on *Sporobolus indicus*.

#### *Hoplandrothrips pergandei* Hinds

A single female was collected from a Valencia orange fruit on May 26, 1995 in Mixom II (Table 2).

### Idolothripinae

A single larva was collected from citrus twigs on September 19 at Hart I (Table 2).

#### *Merothrips floridensis* Watson

This species was collected from trunk scrapings in Trask, Pollard, and Yarborough between January and March 1995 (Table 2). A single female was collected from a fruit of ‘Marsh’ grapefruit on May 22, 1995 in Pollard.

#### *Merothrips morgani* Hood

This species was collected from trunk scrapings in February (Table 2).

#### *Neurothrips magnafemoralis* Hinds

This species was collected from citrus fruit between January and March, May, July, and November in Trask, Hart I, Hart II, and Mixom II (Table 2). One adult was collected from trunk scrapings at Hart I in March (Table 2).

#### *Stephanothrips occidentalis* Hood and Williams

One female each was collected from inner leaves at Yarborough and from Valencia orange fruit at Mixom II during May, 1995 (Table 2). One *Stephanothrips* sp. larva was collected from outer leaves in January, 1995 at Trask.

#### *Symphyothrips* sp

One female was collected from citrus twigs on September 19,1995 at Hart I (Table 2).

### Notes

Dr. J.L. Nation, Department of Entomology, University of Florida acted as editor for this paper.
